# Sleep Duration and Metabolic Syndrome among Early Adolescents—A Cross-Sectional Study in Khartoum State, Sudan

**DOI:** 10.3390/ijerph20095696

**Published:** 2023-05-01

**Authors:** Fatima A. Elfaki, Aziza I. G. Mukhayer, Mohamed E. Moukhyer, Rama M. Chandika, Stef P. J. Kremers

**Affiliations:** 1 Department of Clinical Nutrition, Faculty of Applied Medical Sciences, Jazan University, Jazan P.O. Box 114, Saudi Arabia; 2 School of Nutrition and Translation Research in Metabolism, Maastricht University, 6211 LK Maastricht, The Netherlands; 3 Department of Health Education and Promotion, Maastricht University, 6211 LK Maastricht, The Netherlands; 4 School of Medicine, Ahfad University for Women, Omdurman P.O. Box 167, Sudan; 5 Department of Emergency Medical Services, Faculty of Applied Medical Sciences, Jazan University, Jazan P.O. Box 114, Saudi Arabia; 6 Public Health Programs, School of Medicine, University of Limerick, V94 T9PX Limerick, Ireland

**Keywords:** metabolic syndrome (MetS), sleep duration, adolescents, logistic regression, generalized additive model (GAM), Sudan

## Abstract

Numerous studies have reported that sleep disorders are linked to poor health outcomes. However, studies on these associations in children and adolescents in an African context are limited. The aim of the present study was to analyze the relationship between sleep duration and the presence of metabolic syndrome among early adolescents in Sudan. **Methods:** A cross-sectional study was conducted on participants aged 10–15 years in Khartoum State, Sudan. Metabolic syndrome (MetS) was diagnosed by increased waist circumference and the presence of two or more metabolic abnormalities (triglycerides [TG], high-density lipoproteins [HDL-C], blood pressure [BP], and fasting plasma glucose [FBG]). Short sleep duration was defined based on National Sleep Foundation (NSF) classification. Data were collected by physical examinations, biochemical analyses, and self-developed standardized questionnaires. Data were analyzed with IBM SPSS Statistics Version 24. A generalized additive model (GAM) was used for the smoothing function between sleep duration and MetS. *p* < 0.05 was considered as significant. **Results:** The prevalence of MetS and short sleep among early adolescents aged 10–15 years in Sudan was 2.3% and 55.0%, respectively. A higher prevalence of short sleep was found among overweight and obese participants (*p* < 0.05). The prevalence of MetS among short sleepers was 2.8%. Binary logistic regression analysis showed that male short sleepers had higher odds of having MetS compared to female short sleepers. The relationship between short sleep and low HDL-C in boys and between short sleep and high TG in girls was statistically significant. The highest risk of MetS was observed at less than 6.5 h of sleep per night. **Conclusions:** Short sleep duration was significantly associated with overweight/obesity in the total population and with low HDL-C in boys and high triglycerides in girls. A nonlinear curve pattern was observed between sleep duration and prevalence of MetS. Longitudinal studies are needed to further determine the causal relationship between sleep habits and MetS and its components.

## 1. Introduction

Adolescence is the period between childhood and adulthood. Substantial changes happen during this period in physical, emotional, sexual, and social development [1,2].

Adolescence is a crucial stage in a person’s cognitive and biological growth. Teens have a greater tendency to make hasty decisions, have fragile emotions, and engage in risky activity, among other things [3]. Adequate sleep is vital during this period, and it is an essential indicator of health and quality of life [1,4]. Teenagers’ lack of sleep has a variety of causes, including the rapid growth of technology in society and rising expectations for productivity and education. As a result, adolescents must deal with a variety of stimuli competing for their time and attention every day. Teenagers’ excessive use of gadgets and early start times of school are two specific sources of sleep disturbance and obesity risk. As society modernizes, teenagers and adults have substantially increased their screen usage, especially at night and through social media, which might influence sleep [3]. At least 89% of United States (US) teens use mobile electronic devices during bedtime [5], negatively affecting their sleep. More than two thirds of high school students in the US have been found to sleep less than 8 h per night [6]. Irregular sleep patterns in adolescents, in particular short sleep intervals, can interfere with biological systems’ circadian rhythms [7,8,9]. Teenagers also experience stress and sleep deprivation as scholastic responsibilities rise; these issues may be made worse by early start times of school. Teenagers, who are going through a crucial stage in their physical development, are severely harmed by this. Teenagers also have a natural tendency to sleep later due to a typical sleep phase delay. The difference in sleep length between school days (weekdays) and non-school days (weekends) has been found to be associated with both mental health outcomes and physical health [10]. Those with less sleep variability have a lower risk of hypertension [11,12], obesity [13], and overall mortality [14]. All aspects of adolescent functioning, including academic, psychological, and behavioral outcomes, are impacted by insufficient sleep and sleepiness. For the health and functioning of teenagers, it is crucial to recognize and prevent sleep deficiency and sleepiness [15]. A study from Germany on sleep duration and its relationship with childhood obesity stated an inverse association between sleep duration and fat mass [3]. There is accumulating evidence that short sleep increases the risk of overweight/obesity in children and adolescents, and it is associated with many comorbidities, including type 2 diabetes, hypertension, coronary heart disease, dyslipidemia, and endothelial dysfunction [16,17,18]. 

Both sleep disturbances and cardiometabolic diseases have a high prevalence among African descent populations [19]. The mixture of poor lifestyle, physical inactivity, and short sleep had a strong association with cardiovascular health complications in at-risk youth [20].

Studies from Sudan reported that medical students are prone to sleep disorders and an associated poor academic performance [21,22]. A multinational survey including Sudan stated that more than two thirds of medical students suffer from high internet addiction, which is significantly associated with poor sleep quality [23]. 

Metabolic syndrome (MetS) is a group of metabolic abnormalities that include abdominal obesity, insulin resistance, elevated blood pressure, high levels of triglycerides, and low levels of high-density lipoproteins [24,25]. A growing number of studies from developing countries, including those in South Asia [26,27] and Western Asia [28,29,30,31,32,33], and developed countries [34,35] have identified an increasing prevalence of MetS. Epidemiological evidence from Sudan indicates a 7.5–8.4% prevalence of MetS among Sudanese university students [36,37]. Sleep duration has been recognized as a risk factor for metabolic syndrome [16,38,39,40,41]. A cross-sectional study [42], familial studies [43,44], and cohort studies [45,46] have reported that short sleep duration is associated with higher chances of developing MetS symptoms in the future. Considering the importance of sleep for health, particularly during early adolescence, and because of the scarcity of published data on sleeping patterns and metabolic syndrome in Sudan, the present study aimed to assess the association between sleep duration and metabolic syndrome among early adolescents in Sudan.

## 2. Materials and Methods

### 2.1. Study Design and Population

A descriptive cross-sectional study was conducted, and data were gathered between 2018 and 2019 from primary schools in Khartoum State. Two localities out of seven from Khartoum State were selected by ballot, Khartoum City to represent the urban areas and Karari to represent the rural areas. Three-stage cluster sampling, proportionate to population size, and systematic sampling techniques were used. In total, 21 primary schools including 12 urban and 9 rural were selected. In the first step, a total of 1064 students from the selected schools were asked to fill out the standardized questionnaire related to their socio-demographics: gender, age, residence, parents’ educational level, and how long they usually slept during the night. Prior to data collection, qualified trainers provided a training workshop for all data collectors. The standardized techniques for taking anthropometric, blood pressure, and biochemical measurements were presented in this workshop. In the presence of the researcher and as part of the training, the interviewers were asked to speak with a few students in order to pretest the questionnaire. The procedure for utilizing weighing scales was demonstrated to the teams, emphasizing the need to zero the scale with the scale screw prior to each measurement as well as the need to take off bulky clothing and shoes when taking measurements.

To protect their privacy, participants were given unique identifying numbers. The participants’ consent status was verified by the identifying numbers, which also connected the pupils to their specific schools, clinical assessments, and blood samples. All respondents’ identities were kept completely secret, and only aggregate statistics were reported and made public.

A sensitization program was conducted for health professionals to collect anthropometric measurements, blood pressure readings, and biochemical tests. After the removal of participants with missing information, 921 early adolescents aged 10–15 years were included in the study. The study was approved by the Research Ethics Committee of the Federal Ministry of Health, Sudan (Ref. No. 0001 date 1 December 2017). Written informed consent was obtained from both parents and pupils to participate in the study. 

### 2.2. Physical Examination

Health Professionals conducted physical examinations in accordance with a set process. A digital scale was used to measure weight (SECA with light clothing and without shoes). A handheld stadiometer was used to measure the participants’ height while they were standing barefoot. BMI was converted into a Z-score to determine the prevalence of overweight and obesity. Based on sex and age-specific BMI standard reference points given by the WHO [47,48], obesity was classified as greater than +2SD, underweight as less than −2SD, and overweight as greater than +1SD. Waist circumference (WC) was measured at the midpoint between the bottom of the rib cage and the region above the top of the iliac crest by using an unstretched measuring tape and converted to centimeters (cm), to the nearest 0.1 cm. WC 90th percentile was used to identify abdominal obesity. An automatic digital sphygmomanometer with an adjustable cuff was used to measure blood pressure. To achieve relaxation and blood pressure stabilization, blood pressure was measured twice and the average value was taken.

### 2.3. Biochemical Analysis

Using standardized tubes—a lithium heparin tube for the lipid profile and a fluoride oxalate tube for fasting blood glucose—a laboratory technician collected 5 mL of venous blood from participants (after an overnight fast for at least 8 h). Collected samples were then centrifuged (L500 Tabletop Low Speed Centrifuge) for 10 min at 3000 rpm, giving the blood time to coagulate. The Almogran University Hospital’s laboratory received blood samples. To make sure the analytical testing was accurate, all quality checks were carried out. Serum samples were calculated using the enzymatic method automated by an A25 Analyzer to determine triglycerides (TG) and high-density lipoprotein cholesterol (HDL-C). Using automated equipment, the A25 Analyzer, glycemia was tested using the enzymatic glucose oxidase method to evaluate fasting blood glucose levels. An A25 Biosystems SA Costa Brava 30, Barcelona (Spain) Analyzer was used to evaluate fasting blood glucose, high-density lipoprotein cholesterol, and triglycerides utilizing the required standard laboratory reagents, enzymatic methods, and calorimetric methods. The conventional enzymatic kit approach was used to measure serum triglycerides, fasting blood sugar, and high-density lipoprotein cholesterol.

### 2.4. Definition of Metabolic Syndrome (MetS)

MetS was defined based on the International Diabetes Federation (IDF) guidelines [49]. It is diagnosed by abdominal obesity (waist circumference ≥ 90th percentile) and the presence of two or more other clinical features (triglycerides [TG] ≥ 150 mg/dL, HDL-C < 40 mg/dL, systolic blood pressure [BP] ≥ 130 mmHg or diastolic BP ≥ 85 mmHg, and fasting plasma glucose [FBG] ≥ 100 mg/dL).

### 2.5. Definition of Sleep Duration

Sleep duration was reported by the participants on how long on average they usually slept during the night in the past six months. The National Sleep Foundation (NSF) classification of sleep duration cut-offs based on age [50] was used. For children aged 6–13 years, short sleep is defined as <9 h and normal sleep as 9–11 h. For adolescents aged 14–17 years, short sleep is <8 h and normal sleep is 8–10 h [50]. In total, 4 participants reported exceeding the recommended sleep duration. For the purpose of answering the research question of our study, these participants were not included in the analyses.

### 2.6. Statistical Analysis

Descriptive measures were presented as mean and standard deviations, and categorical variables as frequencies and percentages. Associations between categorical variables with sleep duration were tested with chi-square tests. Logistic regression analyses were applied to determine the odds ratio and its 95% confidence interval for the association between MetS and its components with short sleep, while correcting for the confounding variables including age, father’s educational level, and weight status. The generalized additive model is a generalized linear model in which relationships between predictor and dependent variables follow smooth patterns that can be either linear or nonlinear [51,52]. MetS (yes/no) was used as a binary outcome variable and a smoothing function of sleep duration as a univariable predictor. *p* < 0.05 was considered as statistically significant. Data were analyzed with IBM SPSS Statistics Version 27 (Chicago, IL, USA).

## 3. Results

### 3.1. General Background Characteristics of the Study Population

In total, 921 early adolescents participated in the study, of whom 388 (42.1%) were boys and 533 (57.9%) were girls. More than half of the participants (55.0%) had short sleep duration. Age had a significant association with sleep duration (*p* < 0.01). More than two thirds of the participants in the age group 10–13 years were defined as short sleepers, whereas in the 14–15 years age group, approximately one third were short sleepers. More than half of the early adolescents from both urban and rural areas had short sleep. 

Fathers’ education level had a highly significant positive association with sleep duration (*p* < 0.01); nearly two thirds of the participants (60.8%) whose fathers’ level of education was between university and postgraduate had short sleep duration. A higher percentage of overweight/obese adolescents had short sleep, compared to those with a normal weight or who were underweight [Table 1].

### 3.2. MetS and Its Components’ Association with Short Sleep Duration

Mean differences in MetS components between normal and short sleepers are shown in Table 2. Among short sleepers, 2.8% had MetS, and 1.7% of the normal sleepers had MetS. Early adolescents with short sleep were more likely to display undesirable levels of all MetS components, although they were statistically non-significant.

### 3.3. MetS and Its Components’ Association with Short Sleep Duration Stratified by Gender

Table 3 shows the association between MetS and its components with sleep duration among boys and girls. When comparing the normal sleep early adolescents with those who got short sleep, boys and girls showed similar patterns. The relationship between short sleep and low HDL-C in boys and between short sleep and high TG in girls was statistically significant. 

**Figure 1:**
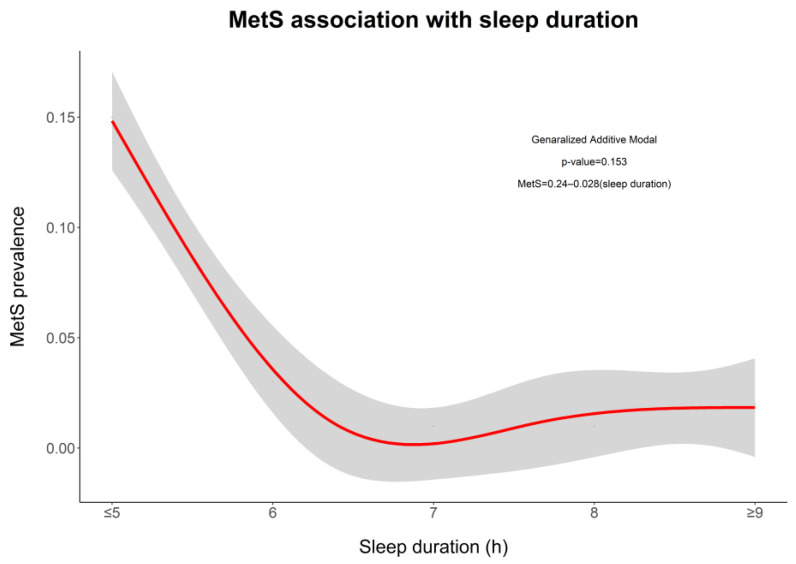
Association between MetS with sleep duration.

Figure 1 shows the nonlinear relationship between MetS and sleep duration in the univariate generalized additive model (GAM). The highest risk was observed among those who sleep less than 6.5 h per night.

## 4. Discussion

This cross-sectional study among early adolescents in Khartoum State, Sudan, showed a trend for components of metabolic syndrome to be associated with short sleep duration and that weight status was significantly associated with short sleep. Evidence regarding an association between sleep duration and MetS in children and adolescents is sparse [53], but evidence is accumulating. Our study adds to the evidence base because studies on this topic in the African region are rare. 

Sleep duration had a significant association with age. The current study found that the 10–13 years age group had a high prevalence of short sleep compared to the age group 14–15 years. Similar results were recorded in early adolescent girls belonging to the age group 11–14 years [54]. In our study, 37.91% of the participants in the age group 10–13 years met the NSF recommended guidelines. In the same vein, a multinational cross-sectional study across five major continents of the world (Europe, Africa, the Americas, South-East Asia, and the Western Pacific) reported that 42% of 9–11-year-old children met the recommended sleep hours according to the Canadian Guidelines [55,56,57]. The reasons for these results may include that substantiated sleep durations vary systematically by sex and race/ethnicity, socio-economic and cultural practices, and age. The present study found that the early adolescents aged 10–15 years whose fathers’ education was at university or postgraduate levels had a high risk of short sleep, which contrasts with two familial studies from Korea that found children whose fathers had a high education level had a low risk of short sleep [58,59]. Since schools and society are imposing so much pressure on children, this might be even more prevalent in Sudanese families with highly educated parents. The varying results may be due to the effect of sample size and social, economic, cultural, and geographical reasons. 

The current study observed that a higher percentage of overweight/obese adolescents had short sleep compared to normal weight or underweight adolescents. A study from China also showed that high BMI was significantly associated with shorter sleep duration [38]. A cross-sectional study from Germany reported the existence of a negative association between sleep duration and body mass index (BMI) [60], and a study in Colombia found that children and adolescents with shorter sleep had higher values of HDL-C [16]. Another study from China reported that early adolescents with shorter sleep had higher mean values of WC, SBP, and TG [38]. In accordance with our findings, various studies in a pediatric population reported that shorter sleep duration was associated with a higher risk of MetS and high WC among Asian, European, and American children and adolescents [16,38,39,41,59,61,62,63]. Another prospective cohort study in Korea showed that short sleep duration (<8 h) was associated with cardiovascular disease (CVD) risk factor clustering [41]. A study on cardiometabolic risk factors in adults analyzed the association between MetS, sleep, and physical activity and reported that undesirable lifestyles and behavioral factors have a risk of MetS in youth [20]. A cross-sectional study in Massachusetts among adolescents aged 11.9–16.6 years demonstrated that each increment of sleep duration (55 min per day) was associated with 2.81 cm decrease in WC [38,40,64]. However, the Nutrition and Health Study from Europe among adolescents stated that sleep duration is not associated with cardiometabolic risk factors when accelerometer-measured physical activity was considered [65]. Our results showed that the early adolescents with less than 6.5 h of sleep per night had a high risk of MetS and 6.5–7 h of sleep per night had the lowest risk of MetS, which is in accordance with a cross-sectional study among the adult population that found the lowest risk of MetS with 7–7.5 h of nightly sleep [51].

Several mechanisms may explain the relationship between short sleep duration and MetS and weight status. Sleep deprivation is associated with more time spent eating, decreased energy expenditure, and less physical activity [66,67,68]. Short sleep duration can also change hormone levels of leptin, ghrelin, insulin, and cortisol, which can lead to an increase in appetite and a preference for energy-dense foods [66,67,69]. In addition, a short sleep duration may contribute to sympathovagal imbalance, which can lead to the development of abdominal obesity [66,67,70].

One of the limitations of the current study was the cross-sectional design, which does not allow for drawing cause and effect conclusions between sleep duration and MetS. Secondly, our measurement of sleep duration did not cater for the difference of sleep duration between school days (weekdays) and non-school days (weekends) and weekend catch-up sleep, which might have provided a clearer picture of sleep duration. Thirdly, sleep duration may not be enough to assess the effect of sleep among adolescents. Questionnaire items regarding sleepiness or restorative sleep would have been useful additional parameters to assess the sleep among adolescents. Further longitudinal research with catch-up sleep, sleepiness, restorative sleep variables with a large sample of all states is needed to assess the sleep effect among adolescents. 

## 5. Conclusions

To the best of our knowledge, this is the first study to examine the relationship between sleep duration and metabolic syndrome among early adolescents in Sudan. The results showed a trend for components of metabolic syndrome to be associated with short sleep duration. In addition, weight status was found to be significantly associated with short sleep. Promotion of healthy sleeping patterns in Sudan is advised to be incorporated into efforts to reduce the burdens of metabolic syndrome and obesity. 

## Figures and Tables

**Table 1:** Association between demographic characteristics and sleep duration.

Variables	*n* (%) 921 (100%)	Sleep Duration		^¥^ *p* Value
		Normal Sleep 414 (45.0%)	Short Sleep 507 (55.0%)	
Gender				
Boys	388 (42.1%)	182 (46.9%)	206 (53.1%)	0.309
Girls	533 (57.9%)	231 (43.5%)	301 (56.5%)	
Age group				
10–13 years	678 (73.6%)	257 (37.91%)	421 (62.09%)	<0.001 *
14–15 years	243 (26.4%)	157 (64.6%)	86 (35.4%)	
Type of residence				
Urban	495 (53.7%)	220 (44.4%)	275 (55.6%)	0.739
Rural	426 (46.3%)	194 (45.5%)	232 (54.5%)	
Father educational level				
Illiterate	60 (6.5%)	30 (50.0%)	30 (50.0%)	0.005 *
Primary	102 (11.1%)	54 (52.9%)	48 (47.1%)	
Intermediate	44 (4.8%)	29 (65.9%)	15 (34.1%)	
Secondary	161 (17.5%)	69 (42.9%)	92 (57.1%)	
University/Postgraduate	380 (41.1%)	149 (39.2%)	231 (60.8%)	
Don’t know	174 (18.9%)	83 (47.7%)	91 (52.3%)	
Mother educational level				
Illiterate	85 (9.2%)	47 (55.3%)	38 (44.7%)	0.295
Primary	160 (17.4%)	75 (46.9%)	85 (53.1%)	
Intermediate	52 (5.6%)	25 (48.1%)	27 (51.9%)	
Secondary	217 (23.6%)	95 (43.8%)	122 (56.2%)	
University/Postgraduate	302 (32.8%)	124 (41.1%)	178 (58.9%)	
Don’t know	105 (11.4%)	48 (45.7%)	57 (54.3%)	
Weight status				
Underweight	153 (16.6%)	75 (49.0%)	78 (51.0%)	0.019 **
Normal	573 (62.2%)	269 (46.9%)	304 (53.1%)	
Overweight	108 (11.7%)	43 (39.8%)	65 (60.2%)	
Obese	87 (9.4%)	27 (31.0%)	60 (69.0%)	

**^¥^** Chi-Square, ** Significant, and * Highly significant.

**Table 2:** Association between short sleep duration and MetS and its components.

Component	Normal Sleep (*n* = 414)	Short Sleep (*n* = 507)	OR (95% CI)	^¥^ *p*-Value
MetS	7 (1.7%)	14 (2.8%)	1.651 (0.660–4.130)	0.284
Mean (Standard deviation)				
WC (cm)	68.61 (10.60)	69.46 (11.17)	1.550 (0.966–2.486)	0.069
SBP (mmHg)	110.98 (11.44)	110.51 (10.87)	1.035 (0.559–1.917)	0.913
DBP (mmHg)	75.37 (11.16)	74.04 (10.74)	1.011 (0.734–1.391)	0.948
FBG (mmol/L)	102.51 (11.41)	102.87 (11.49)	1.128 (0.858–1.483)	0.390
TG (mmol/L)	96.38 (33.53)	97.63 (33.61)	1.016 (0.604–1.874)	0.108
HDL-C (mmol/L)	51.85 (12.50)	52.14 (12.49)	1.375 (0.949–1.992)	0.092

^¥^ Wald test, MetS—Metabolic syndrome, WC—Waist Circumference, SBP—Systolic Blood Pressure, DBP—Diastolic Blood Pressure, FBG—Fasting Blood Glucose, TG—Triglyceride, and HDL-C—High-Density Lipoprotein Cholesterol.

**Table 3:** Association of sleep duration with MetS and its components stratified by gender.

Outcome	Boys			Girls		
	Normal Sleep	Short Sleep	^¥^ *p*-Value	Normal Sleep	Short Sleep	^¥^ *p*-Value
Total						
MetS	1	2.033	0.245	1	1.289	0.730
High WC	1	1.058	0.897	1	1.987	0.067
Elevated BP	1	1.154	0.744	1	1.265	0.624
High TG	1	1.026	0.966	1	2.102	0.019 *
High FBG	1	1.266	0.357	1	1.053	0.782
Low HDL-C	1	1.957	0.034 *	1	1.188	0.499

^¥^ Wald test, * Significant, MetS—Metabolic Syndrome, WC—Waist Circumference, BP—Blood Pressure, TG—Triglyceride, FBG—Fasting Blood Glucose, and HDL-C—High-Density Lipoprotein Cholesterol.
